# Application of Probiotics in Cats and Dogs: Benefits and Mechanisms

**DOI:** 10.3390/vetsci12101008

**Published:** 2025-10-17

**Authors:** Jintao Sun, Xinshu Gu, Huaiyu Zhang, Lihong Zhao, Jinquan Wang, Xiumin Wang, Hui Tao, Zhenlong Wang, Bing Han

**Affiliations:** 1 Key Laboratory of Feed Biotechnology of Ministry of Agriculture and Rural Affairs, Institute of Feed Research, Chinese Academy of Agricultural Sciences, No. 12 Zhong Guan Cun South Street, Haidian District, Beijing 100081, China; 2 College of Animal Science and Technology, China Agricultural University, Beijing 100083, China

**Keywords:** probiotics, cats, dogs, nutrient metabolism, gut health

## Abstract

**Simple Summary:**

This review outlines the benefits of probiotics for dogs and cats, covering their effects on gut health, nutrient metabolism, digestibility, antiviral capabilities, and other clinical conditions. Additionally, it explores the potential mechanisms underlying probiotic applications, which include alleviating intestinal inflammation, enhancing immunity, boosting antioxidant capacity, promoting beneficial intestinal flora, and improving intestinal health.

**Abstract:**

Probiotics have grown increasingly pivotal for the health of pets, particularly dogs and cats. Emerging research demonstrates that probiotics exert a significant positive impact on gut health, including alleviating intestinal inflammation, regulating gut microbiota balance, and relieving diarrhea symptoms for pets. Regarding nutrient metabolism, probiotics aid in prevention and management of obesity and associated metabolic diseases, primarily by enhancing nutrient digestibility and regulating energy utilization and fat metabolism. Furthermore, probiotics exhibit positive effects, including antiviral activity, immune regulation, and antioxidation. Specific probiotic strains exert their functions via mechanisms such as increasing immunoglobulin levels, suppressing the expression of inflammatory factors, and boosting antioxidant enzyme activity. The underlying mechanisms primarily involve regulating metabolites (e.g., short-chain fatty acids, SCFAs), strengthening the intestinal barrier function, modulating immune responses, and optimizing the gut microbial composition. While existing studies highlight the broad potential of probiotics in preventing and managing various chronic diseases in dogs and cats, their utility in addressing acute illnesses and severe organ damage remains limited. Future research should prioritize investigating species-specific mechanisms of actions and extend to exploring potential applications in the neurological health and behavior of pets.

## 1. Introduction

Probiotics are a crucial class of functional live microorganisms known for their ability to enhance intestinal health. In recent years, as pet health has gained growing attention, probiotics have been increasingly utilized to improve gut health of pets, especially dogs and cats. Probiotics exert their effects by improving the gut microbiota balance, strengthening the mucosal barrier, reducing inflammation, lowering intestinal permeability, and positively modulating various aspects of nutrition metabolism [1,2,3].

Notably, certain species of probiotics have been extensively applied to promote the health of dogs and cats, including *Bifidobacterium* spp., *Lactobacillus* spp., *Bacillus* spp., and yeast species, which have been widely recognized and applied in pets. These strains exert beneficial effects through synergistic actions [4]. Additionally, probiotics play a role in preventing and treating other clinical diseases in dogs and cats.

This article summarizes the applications of probiotics in pets in recent years, focusing on gut health, nutrient digestibility and metabolism, and other clinical diseases. It systematically reviews the interrelationship between probiotics and indicators such as intestinal diseases, inflammation, and immunity in dogs and cats, while also revealing the probable working mechanisms.

## 2. Benefits of Probiotics on Pets

### 2.1. Gut Health

Gut health is very important for pets. Clinically, gut problems manifested as enteritis, pancreatitis, gastritis, diarrhea, constipation, inflammatory bowel disease (IBD), and so on, for which nutrition was one of the most important factors affecting gut health. Some of the diseases, like IBD, were lacking of therapeutic drugs, and even if they had them, the drugs could probably cause heavy side effects in pets. Therefore, the veterinarian often prescribes probiotics for curing gut diseases, as they have few side effects compared with drugs. The beneficial impact of probiotics on the intestinal microbiota of dogs and cats is multifaceted and direct. Table 1 showed numerous studies that had highlighted the positive roles of *Lactobacillus* spp., *Bifidobacterium* spp., yeasts, and their derivatives in promoting intestinal health in dogs and cats [5].

Probiotics are also effective in improving the imbalance of intestinal flora. They could increase the abundance of beneficial microorganisms such as *Bifidobacterium* spp., while reducing the population of *Escherichia coli* (*E. coli*) in the intestine [6].

**Table 1:** Summary of scientific reports indicating the potential benefits of using probiotics on the gut health of cats and dogs.

Probiotics	Animals	Intestinal Benefits	References
Both *Saccharomyces boulardii* and *Pediococcus acidilactici*	Cat, *n* = 10	Promoted beneficial bacterial colonization, elevated fecal antioxidants, and reduced inflammatory markers.	[5]
*E. coli* Nissle 1917	Dog, *n* = 38	Improved stool consistency and reduced duration of diarrhea.	[7]
*Lactobacillus sakei*	Dog, *n* = 16	Regulated the gut microbiota balance and enhanced metabolic function.	[8]
*B. longum* KACC 91563	Dog, *n* = 12	Enhanced the fecal microbiota and immune response.	[9]
*Bifidobacterium longum* CECT-7347 (heat-treated) combined with Fibersol-2	Cat, *n* = 12	Anti-inflammatory and antioxidant.	[10]
*Saccharomyces cerevisiae*	Dog, *n* = 16	Improved the dysbiosis index, significantly increased the abundances of Bifidobacterium and Turicibacter, and decreased the abundance of Escherichia coli in feces.	[11]
*Enterococcus faecium* Strain SF68	Cat, *n* = 25	Reduced clinical symptoms of vomiting and diarrhea.	[12]

### 2.2. Obesity and Nutrient Metabolism

At present, the obesity rate in dogs and cats is increasing worldwide. Obesity is becoming a serious problem for pets’ health. Long-term intake of excessive nutrition for dogs and cats without sufficient exercise could lead to a large amount of fat accumulation and even to metabolic disorders and a series of obesity diseases, such as type II; diabetes mellitus, hyperlipidemia, hypertension, and cardiovascular diseases [13]. In particular, metabolic diseases are more prominent in older and obese dogs and cats.

It was proven that obesity was related to the composition of gut microbiota [14]. The composition of gut microbiota could affect the ability of an animal to obtain energy and regulate energy use. For cats, the abundances of *Bifidobacteriaceae*, *Coriobacteriaceae*, and *Veillonellaceae* were significantly higher in the obese versus non-obese group [15]. For obese dogs, the relative abundance of *Faecalibacterium*, *Phascolarctobacterium*, *Megamonas*, *Bacteroides*, *Mucispirillum*, and *Ruminococcaceae* was higher [16]. Low-fat and high-fiber diets can increase the biodiversity of microbial genera and metabolic pathways. Administering probiotics to dogs and cats can change the abundance of gut microbiota, restore the stability of fecal microorganisms, enhance energy utilization, prevent lipid accumulation, and activate pyruvate metabolism in the body [17]. As shown in Table 2, probiotics, mainly *Lactobacillus* spp. and *Enterococcus faecium*, are beneficial in regulating obesity or fat metabolism in pets. Similar studies in cats are limited. Most of the studies showed positive effects, which showed that probiotics could be a proper supplementation in managing lipid metabolism of pets, but it was also indicated that probiotics may have no a significant effect on the health of pets [18], which may indicate that certain species could play a role in nutrient metabolism.

### 2.3. Nutrient Digestibility

Nutrient digestibility is an important parameter when evaluating the quality [24] and bioavailability of pet food. It was shown that cats usually have a lower fat digestibility in comparison to dogs [25]. Because dogs are omnivores, while cats are obligate carnivores, it is valuable to know how well they digest protein from plants and animals. Moreover, there are a number of factors, such as dietary protein content, amino acid composition, and efficiency of dietary protein utilization, that can vary depending on the protein sources in both species [26]. Cats often have a higher protein digestibility compared to dogs, and in dogs, there is not much difference between plant and animal proteins. However, in cats, it was shown that protein from plants was easier to digest than animal proteins [27]. Small cats, including domestic cats, frequently lick and groom, resulting in a large amount of hair ingestion. Therefore, the apparent digestibility of nutrients in dehaired feces was significantly higher than that in feces with hair [28].

Probiotics could improve the growth performance and nutrient digestibility of pets. One of the main reasons for improvement of nutrient digestibility due to probiotics is mainly attributed to the activity of microbial enzymes in the intestinal lumen, including α-amylase, α-galactosidase, cellulase, protease, and lipase [29,30]. The most widely used probiotics in dogs and cats include *Lactobacillus*, *Bifidobacterium,* and *Enterococci* [31]. As shown in Table 3, some studies have indicated that lactic acid bacteria can potentially improve digestibility, as well as enhance stool scores and reduce ammonia in dogs [32,33]. Canine-derived *Lactobacillus Johnson* CPN23 and dairy-derived *Lactobacillus acidophilus* NCDC15 were shown to improve the digestibility of canine fiber (*p* = 0.034) [33]. Feeding cats with *Lactobacillus* increased crude protein digestibility, improved nutrient digestion, and reduced fecal odors [34].

However, spore-forming strains, such as *Bacillus* spp., have been explored as candidate strains for probiotic addition due to the tendency of lactic acid bacteria to lose their activity during processing, storage, and gastrointestinal action [35]. When adult beagle dogs were supplemented with *Bacillus subtilis* C-3102, there was a trend towards higher apparent digestibility of crude fat and nitrogen-free extracts in the supplemented group compared to the diet treatment group without probiotics, as well as higher digestibility of dry matter and organic matter [36]. The study found that *Bacillus coagulans* had an effect on the nutritional digestibility of healthy dogs, which increased the apparent digestibility of organic matter, crude protein, crude fat, and total energy in dogs [37]. Despite the use of *Bacillus* showing beneficial effects on digestibility in dogs, it is currently unclear what should be the minimum dose established for this particular agent [38]. The addition of *Bacillus amyloidis* SC06 and *Bacillus subtilis* B10 at the same time can improve the apparent digestibility of nutrients in the diet of cats [39].

When evaluating nutrient digestibility, odorous substances could sometimes be studied, such as benzpyrole, 3-methylindole and even other odorous matter in the feces of pets [23], which could laterally reflect the protein digestibility of the pet food.

**Table 3:** The effects of probiotics on nutrient digestibility in pets.

Probiotics	Animals	Effects	Reference
*Lactobacillus Johnson* CPN23 and *Lactobacillus acidophilus* NCDC15	dogs	Improving the digestibility of fiber.	[33]
*Lactobacillus*	cats	Increasing crude protein digestibility, improving nutrient digestion, and reducing fecal odors.	[34]
*Bacillus subtilis* C-3102	dogs	An upward trend in the apparent digestibility of crude fat and nitrogen-free extracts was observed.	[36]
*Bacillus coagulans*	dogs	Increasing the apparent digestibility of organic matter, crude protein, crude fat, and total energy in dogs.	[37]
*Bacillus amyloidis* SC06 and *Bacillus subtilis* B10	cats	Improving the apparent digestibility of nutrients.	[39]

### 2.4. Clinical Diseases

Except gut-related illnesses, there were less studies on probiotics’ effects on other pet illnesses. Limited studies about chronic kidney disease (CKD) and the application of probiotics in pets existed. From Table 4, it can be concluded that in the CKD trial, the effects of the probiotics were very limited, which probably meant that probiotics could show a benefit in prevention instead of curing. Maybe in the future, more illnesses could be studied in other clinical diseases of pets, especially for elderly pets.

### 2.5. Limitations of Probiotic Application

As mentioned before, not all probiotics showed the positive effects. Mark et al. found in a double-blind controlled clinical trial that probiotics could not alleviate CKD in cats [43]. Although probiotic *Enterococcus faecium* SF68 has been shown to stimulate mucosal immunity and improve intestinal health in young dogs [44], it was found that the bacteria had no significant effects on food intake, body weight, body composition, or metabolic parameters in overweight and obese cats [12]. Short-term treatment with *E. faecium* SF68 in dogs with chronic illnesses did not affect immune responses [12]. These findings indicate that probiotics are not a panacea. In clinical treatment, it is essential to use safety-evaluated probiotics based on their specific characteristics. The field of probiotic safety is characterized by a lack of studies specifically designed to assess safety, making it difficult to evaluate hidden risks associated with probiotic use [45]. More controlled trials are needed to characterize novel, safe probiotic formulations that impact the overall health, well-being, and health maintenance of dogs and cats [46].

## 3. Mechanism

### 3.1. Antivirus

Viruses are one of the most serious factors threatening pets’ health, like FPV (Feline Panleukopenia Virus), FCV (Feline Calicivirus), FHV-1 (Feline herpesvirus 1), and FIP (Feline infectious peritonitis), which could cause low spirits, diarrhea, and even death to pets. As is known, some viruses spread very fast. Although probiotics could not directly kill virus, it could be beneficial in the prevention of virus. It has been preliminarily shown that pre-treatment of FCV with *Lactococcus lactis* subsp. *lactis* LM0230 could result in a reduction in virus titers in vitro [47]. Also, *Enterococcus faecium* SF68 was proven to have a positive effect by lessening the morbidity associated with FHV-1 infection in cats [48]. These studies were limited and did not discover the deep mechanism of antiviral activity of probiotics. But many studies have shown the mechanism of probiotics against antivirus, not for pets but humans or other animals [49,50], which showed that probiotics pose a defensive possibility against respiratory viral infection. The clinical score of dogs with distemper-associated diarrhea could be improved by feeding with *Lactobacillus murinus* native strain (LbP2) for 5 days [51]. But few studies were focused on the mechanism of antiviral ability of probiotics; maybe in the future, this aspect could be investigated more.

### 3.2. Anti-Inflammation

**Figure 1:**
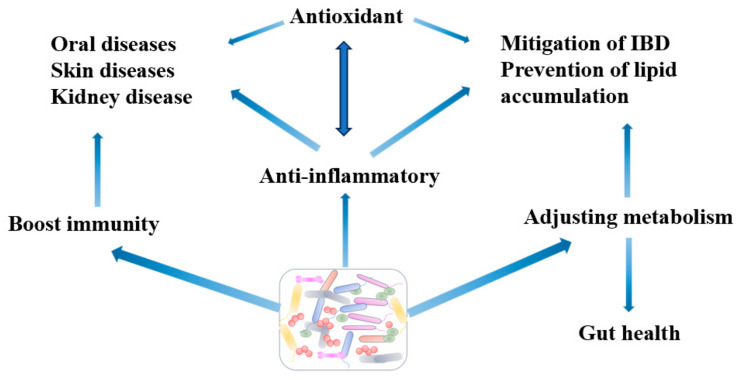
The application of probiotics in pets.

Probiotics can adjust the intestinal flora with a positive impact to alleviate inflammation due to pet diseases. Probiotics mainly work by regulating metabolites (short-chain fatty acids, SCFAs), as well as through the regulation of cell factors and modified mucosa expression of inflammation (shown in Figure 1).

SCFAs have a strong correlation with anti-inflammation effects in inflammatory bowel disease (IBD) in pets, playing an important role in the pathogenesis of the disease [52,53,54,55]. Probiotics could produce SCFAs, mainly acetic acid and propionic acid. SCFAs could inhibit the activity of inflammatory mediators in the intestinal epithelium, thereby inhibiting the activation of NF-κB macrophages [56]. *Bifidobacterium longum* S3 could increase the levels of acetic acid and stearic acid, repair intestinal barrier damage, and reduce inflammation [57]. *Enterococcus faecium* IDCC 2102 and *Bifidobacterium lactis* IDCC 4301 could reduce systemic inflammation and hormonal disorders caused by obesity in dogs by producing short-chain fatty acids and carboxylic acids [17]. Multiple high-concentration strains of probiotics could affect dog colon polyamine biological synthesis and raise the precursor putrescine, as well as spermine and ornithine decarboxylase levels, reflecting anti-inflammatory effects [58]. Synbiotic-IgY supplement could reduce inflammatory markers (fecal calprotectin, C-reactive protein), thereby showing beneficial effects on inflammation and mucosal microbiota in dogs [59].

SCFAs produced by probiotics could protect the intestinal mucosal barrier, inhibit intestinal inflammation, and participate in the prevention of intestinal inflammation [60,61]. SCFAs have anti-inflammatory activities [56], indicating the inhibition of the activity of inflammatory mediators in the intestinal epithelium, thereby inhibiting the activation of NF-κB macrophages. At the same time, SCFAs are also the main energy source for colon cells. Among all SCFAs, butyrate, as the main energy source for intestinal epithelial cells [62], has the strongest anti-inflammatory effect [63]. Currently, one of the key factors contributing to intestinal mucosal inflammation in numerous pathological conditions is energy deficiency. Li [5] found that the addition of *Saccharomyces boulardii* and *Pediococcus acidilactici* can increase the concentration of butyric acid and total SCFA concentration in cat feces, which played a crucial role in maintaining intestinal homeostasis [64]. Similar results were obtained by feeding compound *Bacillus* to cats [65]. Feeding probiotics to dogs has been found to increase the levels of acetic acid and propionic acid, along with the beneficial bacteria in the gut [9].

Many studies have reported that lactic acid bacteria could reduce the expression of inflammatory factors such as IL-6, IL-1β, TNF-α, and IFN-γ, suggesting its potential anti-inflammation mechanisms [66,67,68], but the anti-inflammation mechanisms of probiotics in dog and cat inflammation models are less studied in terms of animal welfare, but it is foreseeable that probiotics have great potential for the treatment of inflammation and prevention in pets.

### 3.3. Immunity

Probiotics could improve immune function, especially in the early life of pets [69]. Both live probiotics and their metabiotics may produce immune effects in pets.

Salvatore [70] found that adding composite probiotic supplements in different pregnancy periods of dogs will enhance the immunity of colostrum, and the content of IgG and IgM in colostrum were significantly increased. It may improve the clinical condition and immune function of puppies [70] and has good clinical effects on preventing gastroenteritis in offspring [71]. *Enterococcus faecium* (SF68) and *Lactobacillus murinus* (LbP2) strains were, respectively, shown to improve the concentration of IgA in dog feces and enhance the specific immunity of the canine [63,72]. It has been reported that adding probiotics to cat food can directly increase the concentration of IgAto, increasing the immune ability of cats [63].

Probiotics can interfere with lymphocytes, especially T cells, which have a strong positive effect. Microbial interactions with Mesenchymal stem cells can increase transcription of key immunomodulatory genes, including COX2, IL6, and IL8, with strong clinical potential for Crohn’s disease, chronic sepsis, and wound healing [73]. A study found that probiotics (VSL#3) can reduce the number of CD3+ lymphocytes of dogs with IBD, while at the same time increasing the FoxP3+ and TGF-β+-positive cell number, maintaining the balance of the immune system [74]. *Bacillus subtilis* strains from fermented soybean could increase the number of peripheral blood NK cells (natural killer cells) and increase the toxic effect of NK cells to enhance cellular immune activity in dogs, and they may cause treatment effects in canine tumor diseases [75]. The expressions of TNF-α, IL-8, and TLR2 were significantly increased in dog macrophages treated with Quilodran-Vega SR. Studies have shown that lactic acid bacteria can inhibit the growth of common Gram-negative bacteria in gastrointestinal infection and regulate the immune response of animals [76]. *Bifidobacteria*-fermented cracking (BFL) can effectively reduce the THP-1 cell macrophages induced by LPS in IL-8, while TNF alpha reduced the secretion of cytokines and cox-2 mRNA expression to adjust the immune balance [77]. Therefore, as in humans, probiotics also play an important role on the immune function in pets, but the mechanisms may need further study.

### 3.4. Antioxidant

Oxidative stress is defined as an imbalance between the occurrence of reactive oxygen species/nitrogen (ROS/RNS) and cellular antioxidant defenses; oxidative stress is the result of an excess of ROS/RNS, which arises due to a lack of reaction by the cellular antioxidant system, which leads to lipid, protein, and DNA damage [78,79]. It plays an important role in a variety of disease states, including chronic kidney disease, neurological disorders, diabetes, obesity, cancer, intestinal inflammation, and diarrhea, among others [80,81,82]. At present, a large number of tests in vitro and in vivo have proven that probiotics have a strong antioxidant effect [83,84]. According to a large number of existing studies, probiotics could regulate the redox state of the host through their metal ion chelating ability, antioxidant system, regulation of signaling pathways, and enzyme-producing ROS [85].

Studies have shown that supplementation with probiotics and postbiotics has no adverse effects on oxidative stress in adult cats [86]. Due to the host specificity of probiotics [87], an increasing number of studies have been focused on isolating probiotics from dogs and cats for the application in dogs and cats. Specifically, *Lactobacillus johnsonii* CPN23 or *Lactobacillus acidophilus* NCDC15 (10^8^ CFU/mL) reduced the levels of glutathione or glutathione S-transferase in probiotic-treated dogs. In contrast, dogs supplemented with *Lactobacillus Johnsonii* CPN230 exhibited higher activities of superoxide dismutase (SOD) and glutathione peroxidase (GPx) [88]. Notably, probiotic LGGs (screened from dog feces) showed a more apparent effect in canine macrophage cell lines (DH82) than RAW264.7 cells, reducing oxidative stress [87]. Relevant studies have indicated that 16 probiotic strains isolated from dogs and cats possessed high antioxidant activity. These strains exert antioxidant activity by inhibiting iNOS and COX-2 gene expression [89].

### 3.5. Adjusting Metabolism and Gut Microbiota Balance

Probiotics can improve the health of dogs and cats by regulating their metabolism and physiological metabolism. Several common metabolites, like SCFAs, EPS, and bacteriocins produced by probiotics, can directly participate in the regulation of signaling pathways to interfere with the metabolism of dogs and cats.

As mentioned before, probiotics could produce many useful metabolites for the host. Therefore, probiotics could modulate intestinal metabolism and maintain intestinal microecological balance by regulating the abundance of gut microbiota. Feeding *Lactobacillus cleicerae* can increase Firmicutes, Spirochaetota, and Patescibacteria at the phylum level, reduce the abundance of Fusobacteriota, Deferribacterota, and Bacteroidota, and down-regulate Anaerostipes, Clostridium_sensu_stricto_13, Parvimonas, Streptobacillus, Mucispirillum, Turicibacter, Fusobacterium, and the levels of multiple pathogenic genera such as Terrisporobacter [88]. Compound probiotics can indirectly increase the pathways of polysaccharide synthesis and metabolism, energy metabolism, immune system metabolism, environmental adaptation, cofactor and vitamin metabolism, and amino acid metabolism by affecting the abundance of intestinal microflora in dogs and reduce the impact of genes related to cell movement, transcription, and membrane transport [90]. Compound *Bacillus* significantly increased the abundance of p_Patescibacter and *g_Plectosphaerella* and decreased the abundance of p_Firmicutes, p_Gemmatimonadetes, *g_Ruminococcaceae*_UCG-005, *g_Ascochytahe,* and *g_Saccharomyces* in cats [65]. Feeding a *Lactobacillus* mixture can reduce gut-derived uremic toxin by regulating amino acid metabolism and achieve the effect of alleviating and treating chronic kidney disease [91].

EPS are particularly beneficial in addressing intestinal health [6]. The intestinal epithelial barrier function in dogs and cats is highly similar to that in humans, forming a selective barrier that absorbs nutrients, water, and other important molecules while preventing the entry of pathogens and harmful substances [92]. Changes in the characteristics of the colonic mucus layer or the reduction in its core component Mucin 2 may lead to bacterial infiltration into the mucus layer and subsequent contact with intestinal epithelial cells, breaking the balance of the intestinal epithelial barrier and then causing inflammatory bowel disease [93]. Probiotics regulate intestinal epithelial barrier function through surface macromolecules and metabolites. The surface-layer proteins (including flagella, fimbriae, and capsular polysaccharide) of probiotics can be recognized by pattern recognition receptors, which play an important role in regulating intestinal barrier function and promoting intestinal health [94]. Probiotics directly or indirectly (via secreted proteins, indole, extracellular vesicles, short-chain fatty acids, and bacteriocins) promote mucus secretion by goblet cells. Increasing the secretion of antimicrobial peptides and increasing the expression of tight junction proteins can protect the intestinal epithelial barrier [95,96]. An imbalance in the normal gut microbiota can increase intestinal permeability, so supplementing with probiotics is an important means of enhancing the body’s immunity by improving gut health and maintaining the function of the intestinal epithelial barrier.

In conclusion, probiotics could play an important role in many functions in pets, but for the protection of animal welfare, there is still little research on the mechanism of the application of probiotics in pets. Maybe more related studies could be carried out, and more functions could be found in pets in the future.

## 4. Conclusions

Studies on the application of probiotics in dogs were widely performed, but research in cats remains limited. It is important to note that probiotics are not a panacea. Probiotics primarily target chronic conditions, demonstrating particularly significant efficacy in regulating gastrointestinal disorders. Currently, probiotics such as lactic acid bacteria and bifidobacteria have been widely adopted in clinical settings. However, when dogs or cats exhibit acute diarrhea, infectious diseases, or severe intestinal organ damage, prompt veterinary intervention with antibiotics or other medications is essential rather than relying solely on probiotics. Long-term excessive use of probiotics may lead to intestinal dysbiosis, so their administration in dogs and cats requires targeted and appropriately dosed regimens.

Currently, due to animal welfare constraints, most mechanistic studies are conducted in mice, and future research should prioritize species-specific investigations in dogs and cats. Research on probiotics for canine and feline health should not be confined to gut health. Similarly, dogs and cats can develop anxiety disorders, depression, Parkinson’s disease, Alzheimer’s-like conditions, and other human-mimicking ailments due to environmental changes, seasonal shifts, owners’ emotional states, or separation from offspring. Therefore, there is vast potential for the application of probiotics in addressing neurological and behavioral health issues in dogs and cats.

## Figures and Tables

**Table 2:** The effects of probiotics on nutrient metabolism.

Probiotics (Dose)	Animals and Duration	Effects	Reference
*Lactobacillus gasseri* BNR17 (2 × 10^9^ CFU/g), *Lactobacillus plantarum* (10^9^ CFU/g)	Five dogs for 10 weeks	Body weight and subcutaneous fat mass were decreased significantly, and microbial diversity was increased.	[19]
*Lactobacillus fermentum* AD1 (10^9^ CFU/g)	Fifteen dogs for 7 days	*Faecal Lactobacilli* and *Enterococci* abundance were increased significantly. Total protein and total lipid were increased.	[20]
*Enterococcus faecium* EE3 (10^9^ CFU/mL)	Eleven dogs for 1 week	Increased fecal Lactic acid bacteria abundance and decreased total lipid and protein levels.	[21]
*Enterococcus faecium* IDCC 2102 (10^10^ CFU/g) and *Bifidobacterium lactis* IDCC 4301 (10^10^ CFU/g)	Twenty dogs for 9 weeks	By restoring fecal microbiota stability, these probiotics enhanced systemic energy utilization and prevented lipid accumulation.	[16]
*Lactobacillus acidophilus*	Twelve dogs for 4 weeks	Serum cholesterol was apparently reduced.	[22]
*Lactobacillus plantarum* L11	Twelve cats for 4 weeks	Serum total cholesterol was decreased, and the abundance of *Bifidobacterium* was improved.	[23]
*Enterococcus faecium strain* SF68	Twenty cats for 8 weeks	No significant effects.	[18]

**Table 4:** Summary of scientific reports exploring the potential effects of using probiotics to counteract different kinds of clinical diseases in pets.

Probiotics	Animals	Clinical Diseases	Effects	Reference
*Lactobacillus acidophilus*	13 dogs	Oral health	Porphyromonas gingivalis was inhibited.	[40]
*Lactobacillus rhamnosus strain* GG	2 adult Beagles with severe AD and 16 puppies	Atopic Dermatitis (AD)	Immunologic indicators were reduced.	[41]
*Probiotics* and *prebiotics*	10 cats with CKD	Chronic kidney disease (CKD)	No significance.	[42]
*Kibow Biotics*	Small number of cats with azotemia	Feline azotemia	BUN was decreased.	[43]

## Data Availability

No new data were created or analyzed in this study.
